# Evaluation of the Effects of Hidden Node Problems in IEEE 802.15.7 Uplink Performance

**DOI:** 10.3390/s16020216

**Published:** 2016-02-06

**Authors:** Carlos Ley-Bosch, Itziar Alonso-González, David Sánchez-Rodríguez, Carlos Ramírez-Casañas

**Affiliations:** 1 Institute for Technological Development and Innovation in Communications, Edificio Polivalente II, 2ªplanta, Parque Científico y Tecnológico, Campus Universitario de Tafira, Las Palmas de Gran Canaria 35017, Spain; david.sanchez@ulpgc.es (D.S.-R.); carlos.ramirez@ulpgc.es (C.R.-C.); 2 Department of Telematic Engineering, University of Las Palmas de Gran Canaria, Campus Universitario de Tafira, Las Palmas de Gran Canaria 35017, Spain

**Keywords:** IEEE802.15.7, VLC, MAC, CSMA/CA, Hidden node problem

## Abstract

In the last few years, the increasing use of LEDs in illumination systems has been conducted due to the emergence of Visible Light Communication (VLC) technologies, in which data communication is performed by transmitting through the visible band of the electromagnetic spectrum. In 2011, the Institute of Electrical and Electronics Engineers (IEEE) published the IEEE 802.15.7 standard for Wireless Personal Area Networks based on VLC. Due to limitations in the coverage of the transmitted signal, wireless networks can suffer from the hidden node problems, when there are nodes in the network whose transmissions are not detected by other nodes. This problem can cause an important degradation in communications when they are made by means of the Carrier Sense Multiple Access with Collision Avoidance (CSMA/CA) access control method, which is used in IEEE 802.15.7 This research work evaluates the effects of the hidden node problem in the performance of the IEEE 802.15.7 standard We implement a simulator and analyze VLC performance in terms of parameters like end-to-end goodput and message loss rate. As part of this research work, a solution to the hidden node problem is proposed, based on the use of idle patterns defined in the standard. Idle patterns are sent by the network coordinator node to communicate to the other nodes that there is an ongoing transmission. The validity of the proposed solution is demonstrated with simulation results.

## 1. Introduction

Optical wireless communications based on visible light [1], named Visible Light Communications (VLCs), use visible light to transmit data by modulating intensity in light emitting diodes (LED), employing faster switching rates than the persistence of the human eye to avoid flickering in data/light sources. In recent years, the increasing use of LEDs in illumination systems has been conducted due to the emergence of the VLC technologies, both in indoor and outdoor environments [2]. In 2011, the IEEE (Institute of Electrical and Electronic Engineers) published the IEEE 802.15.7 standard [3], which defines Physical (PHY) and Medium Access Control (MAC) layers for short-range wireless optical communications using visible light. The MAC protocol plays a significant role in the performance of this standard, and includes a channel access procedure based on the CSMA/CA mechanism [4].

The hidden node problem is a well known problem in wireless networks based on radio frequency using CSMA/CA channel access mechanisms [5,6]. This problem is caused by signal coverage constraints, resulting in network nodes not being able to detect transmissions performed by other nodes. This problem can produce collisions due to concurrent transmissions, potentially degrading the performance of the communications in the network. The use of Resquest to Send/Clear to Send (RTS/CTS) define frames to reduce collisions produced by hidden nodes is a common solution adopted in wireless networks. For example, the IEEE 802.11 standard specification includes the option of using RTS/CTS frames in MAC layer protocol. Regarding the use of RTS/CST frames in IEEE 802.15.4 networks, authors in [7,8,9] have questioned the effectiveness of these solutions for several reasons, including the fact that, during backoff delays time, RTS/CTS frames sent by other nodes are not received because each node transceiver is disabled to reduce energy consumption, which is an important requirement in these type of networks [6]. The same restriction applies to backoff delays in the IEEE 802.15.7 standard.

In order to improve efficiency in energy consumption, the use of directional antennas has been proposed for wireless sensor networks [10]. Directional antennas provide several advantages such as spatial reuse channel and increases in coverage distance. However, directional antennas present a new problem called the directional hidden node problem, which is quite similar to the problem found in VLC networks. Solutions to the the directional hidden node problem in wireless sensor networks have been proposed, see [10], based on the rotation of the directional antenna. The rotation of the antenna is combined with some type of scheduling scheme in order to receive the transmissions coming from surrounding nodes. However, these type of solutions involve significant changes in the standard specification.

In VLC, the hidden node problem can be considered as a key issue because of the directional characteristics of both optical transmitters (LED) and receivers (photo detectors).

Since the IEEE 802.15.7 standard was published in 2011, we are unaware of other studies or published works to evaluate the effects of the hidden node problem when using CSMA/CA mechanisms in a VLC network. Several research papers have been published to evaluate the IEEE 802.15.7 uplink (from user terminals to data/lightning infrastructure) performance based on CSMA/CA. Papers [11,12] presented analytical and simulation models to analyze uplink performance, but their authors stated explicitly that there are not hidden nodes in their proposed models. In [13], the performance of transmissions using a simulation model is discussed, although the authors do not specify the physical channel model used, and they do not indicate if they take into account the presence of hidden nodes.

In this paper, we present an evaluation of the effects of the hidden node problem in the performance of uplink communications when using the slotted CSMA/CA random access procedure defined in the IEEE 802.15.7 standard. Furthermore, in order to improve network performance in the presence of hidden nodes, one solution is presented, which has been chosen not only according to effectiveness and ease of implementation criteria, but also taking into account its ease of integration in IEEE 802.15.7 standard, since it requires no modifications. Our proposed solution is based on selecting the type of idle patterns to be sent by the network coordinator, depending on whether or not it is receiving transmissions coming from network devices. According to IEEE 802.15.7 standard specification, sending idle patterns is a mandatory requirement for infrastructure nodes while idle or receiving operations, to ensure continuous illumination and avoid flickering.

The rest of this paper is organized as follows: Section 2, describes the fundamentals of VLC channel modeling, and the main causes of the hidden node problem are also analyzed. Next, Section 3 provides an overview of the IEEE 802.15.7 standard. In Section 4, we present a description of the simulation model implemented to analyze and evaluate the performance of the CSMA/CA channel access mechanism defined in IEEE 802.15.7. In Section 5, simulation results obtained with and without the presence of hidden nodes are analyzed and compared. In Section 6, a solution to the hidden node problem is proposed, and its effectiveness is analyzed. Finally, Section 7 contains the conclusions of this work.

## 2. Fundamentals of Optical Wireless Communications

Optical wireless links for indoor environments can be classified according to two criteria. The first criterion is the degree of directionality of the optical transmitter and receiver. The second one relates to whether the link relies upon the existence of an uninterrupted line of sight (LOS) path between the transmitter and receiver [14].

**Figure 1:**
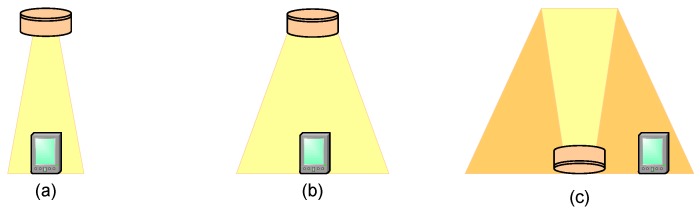
Optical link configurations in VLC: (**a**) directed LOS; (**b**) non directed LOS; (**c**) diffuse.

- Directed links resort to directional transmitters and receivers, which must be aimed in order to establish a link. Directed links maximize power efficiency since path loss and reception of ambient light noise is minimized. They have the disadvantage of limiting mobility in user terminals because they need to be aligned. On the other hand, non directed links may be more convenient to use for mobile terminals, since they do not require precise alignment. However, non directed links present some disadvantages—such as multipath distortion and lower power efficiency—that make it difficult to achieve high transmission rates.
- LOS links require a direct path between the transmitter and receiver that is without obstacles. They allow for maximizing received power and minimizing multipath distortion, but the need for a line of sight diminishes flexibility of use. Non-LOS (NLOS) links are more robust because they can operate even in the presence of obstacles between the transmitter and receiver. NLOS non directed links are referred to as diffuse links. VLCs can use several link configurations, like directed LOS, non directed LOS and diffuse links, see Figure 1 [15].

### 2.1. Channel Model in VLC

**Figure 2:**
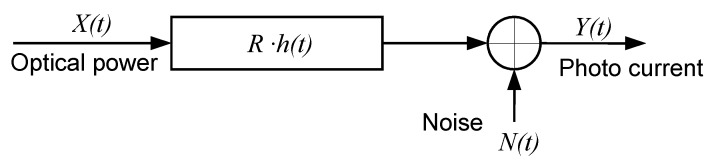
Optical channel modeled as a base band linear system.

The optical channel components are the optical transmitter (LED), the photo detector (PD) and the transmission medium. For VLC links, intensity modulation (IM) is used, in which the waveform of the signal to be transmitted is modulated onto the instantaneous power of the optical carrier. The technique used in reception is direct detection (DD), in which a photodetector produces an electrical current proportional to the received optical instantaneous power. Usually, optical wireless systems based on IM/DD are modeled as a base band linear, time-invariant system [14], see Figure 2.

In Figure 2, X(t) is the instantaneous input power, Y(t) is the output current, and h(t) is the impulse response. N(t) is signal-independent additive noise and *R* is the receiver responsivity. This base band channel model can be expressed by Equation (1), where ⊗ symbol denotes convolution.

(1) Y(t)=R·X(t)⊗h(t)+N(t)

The impulsive response, h(t), is determined by transmitter and receiver characteristics but also depends on their position, orientation and optical signal reflections as well. Plenty of works have been published to characterize an optical wireless channel and its impulsive response h(t), such as [16] based on evaluating of measures, [17] which applies iterative algorithms, or [18] based on statistical methods. Others works have also been published focusing on studying the VLC channel, such as [19,20,21].

#### 2.1.1. Directed LOS Channel Gain

Frequency response of optical channel is relatively flat near Direct Current (DC), so the most important quantity for characterizing this channel is the DC gain H(0) [14], which relates the transmitted and received average power, see Equation (2):

(2) $$P_{r}=H\left(0\right)\cdot P_{t}$$

**Figure 3:**
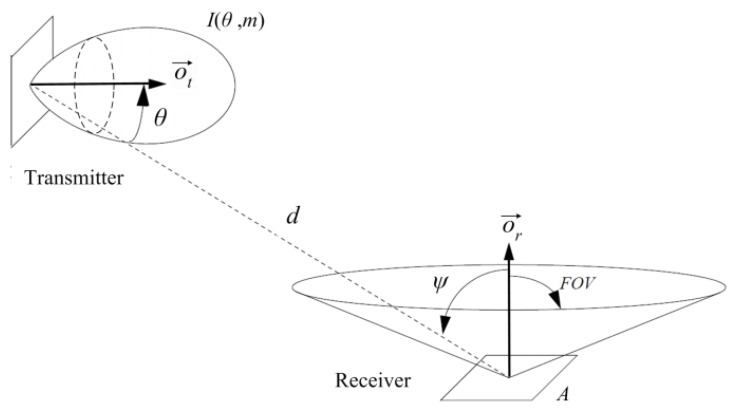
Transmitter and receiver in directed LOS link configuration.

In VLC, the received power can be expressed as the sum of LOS and NLOS components [21]. In directed LOS links, the h(t), hence the DC gain, can be computed fairly accurately by considering only the direct LOS propagation path. Figure 3 shows an example of a directed LOS link. An optical source can be modeled by its position vector, a unit-length orientation vector ${\vec{o}}_{t}$, transmission power $P_{t}$ and a radiation intensity pattern I(θ,m) emitted in direction *θ*. Where *m* is the mode number of the radiation lobe, which specifies the directionality of the source, and is related to the transmitter half power angle $\theta_{1/2}$. Similarly, a receiver is defined by its position, orientation ${\vec{o}}_{r}$, photo detector area *A*, and field of view (FOV). The angle formed between the optical incident signal and the orientation vector ${\vec{o}}_{r}$ is called the incident angle *ψ*. The maximum incident angle defines the receiver FOV.

According to [14], when considering only the direct LOS propagation path, the channel DC gain H(0) is given by Equation (3):

(3) $$H\left(0\right)=\frac{P_{r}}{P_{t}}=\left\{\begin{array}{cc} \frac{m+1}{2\pi \cdot d^{2}}\cdot \cos^{m}\left(\theta \right)\cdot A\cdot G\left(\psi \right)\cdot T\left(\psi \right)\cdot cos\left(\psi \right) & 0<\psi \leq FOV\\ 0 & \psi >FOV \end{array}\right\}$$

In Equation (3), T(ψ) is the signal transmission coefficient of the optical filter in receiver, G(ψ) is the receiver optical concentrator gain, and *d* is the distance between transmitter and receiver. The Equation (3) for channel DC gain in the directed LOS link configuration is based on considering the optical transmitter as a single point source, though VLC transmitters tend to be composed by a large LED array in order to improve illumination capacity. Authors in [22] compare the channel characteristics of both the single point-source model and the array of LEDs. The results obtained show that the deviations are acceptable in terms of the channel optical path loss, as well as bandwidth. There are differences in terms of Root Mean Square (RMS) delay-spread results, though they remain acceptable as long as the LED array is of moderate size.

#### 2.1.2. Receiver Signal to Noise Ratio

Silicon photo-diodes are usually employed in optical receivers to convert the optical signal into an electrical signal, which has an added noise. In VLC systems, there are two main noise sources: thermal noise, $\sigma_{thermal}^{2}$, and shot noise, $\sigma_{shot}^{2}$. Both noise sources can be modeled as additive white Gaussian noise (AWGN) according to [20]. Since both types of noise are uncorrelated, the total added noise can be expressed as the sum of contributions from shot and thermal noise, as expressed in Equation (4):

(4) $$\sigma_{total}^{2}=\sigma_{thermal}^{2}+\sigma_{shot}^{2}$$

Then, the signal to noise ratio (SNR) can be expressed as:

(5) $$SNR=\frac{P_{er}}{\sigma_{total}^{2}}=\frac{R^{2}\cdot P_{r}^{2}}{\sigma_{total}^{2}}$$

In Equation (5), $P_{er}$ is the electrical power of the received signal at receiver’s output, $P_{r}$ is the optical power of the signal received by the photo diode, *R* is the receiver responsivity, and $\sigma_{total}^{2}$ is the total noise power. VLC channel analysis and characterization is an open research line with plenty of contributions, [23,24,25,26]. Published works include studies based on the analysis of aspects such as modulations, codifications, SNR, and Bit Error Rate (BER) among others.

### 2.2. The Hidden Node Problem in VLC Networks

The hidden node problem is a well known problem in radio wireless networks using channel access mechanisms based on contention techniques as CSMA/CA [4]. CSMA/CA based methods are used in most wireless networks such as Wireless LAN (WLAN) [27] or sensor networks [28]. The hidden node problem is caused by signal coverage constraints, resulting in network nodes not being able to detect transmissions performed by other nodes. Thus, a node performing a carrier detection to check the status of the communication channel may detect the channel idle and continue to transmit, leading to possible collisions. Most research has been undertaken to evaluate the effects of this problem in radio frequency based networks, such as [5,6,29,30,31]. In VLC networks, the hidden node problem is also produced in a similar way to radio wireless networks, but, in this case, the problem is further aggravated by the directive characteristics of optical transmitters and receivers.

**Figure 4:**
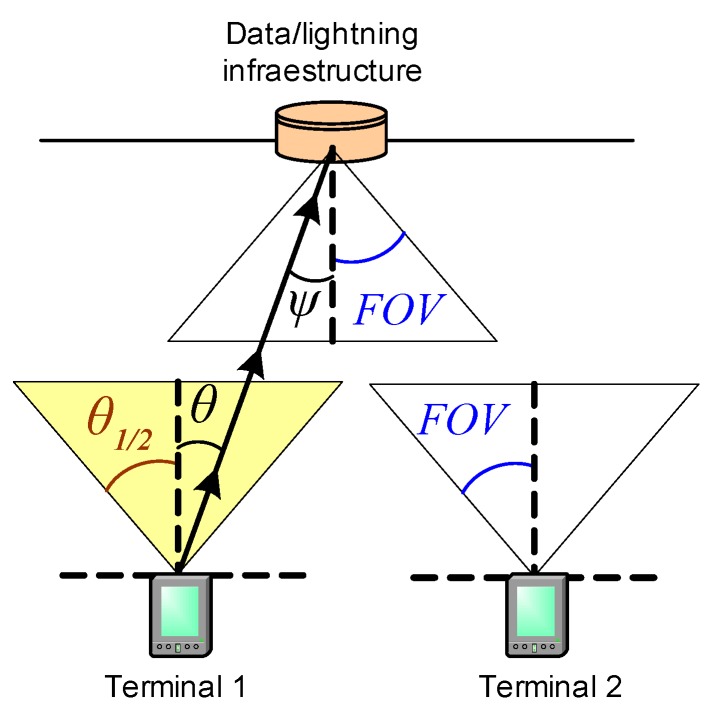
Example of scenario with hidden nodes in a VLC network.

Figure 4 shows a sample scenario of a VLC network which presents the hidden node problem. In this example, two user terminals try to make an uplink transmission to a data/illumination infrastructure device, located at the ceiling of the enclosure. All nodes in the network employ a medium access control procedure based on CSMA/CA method.

In Figure 4, if terminal 2 performs a carrier detect procedure while terminal 1 transmits to the infrastructure, due to the directivity of terminal 1 optical transmitter $\left(\theta_{1/2}\right)$ and terminal 2 optical receiver (FOV), transmitted signal of terminal 1 is not detected by terminal 2. Consequently, terminal 2 will consider the medium is idle and will transmit at the same time terminal 1 is transmitting, thus producing a collision at the infrastructure. IEEE 802.15.7 MAC layer defines a medium access control procedure based on the CSMA/CA method; therefore, uplink transmissions made according to this procedure are susceptible to be affected by the hidden node problem. This situation was revealed in some works published during the standard development [32] and in some documents [33] with proposals to take into consideration by the group responsible of developing the standard. However, in the current version of the standard [3], there are not specific mechanisms designed to solve this problem. To the best of our knowledge, there are not any published works that evaluate the impact of hidden nodes in performance of uplink transmissions made using CSMA/CA in the IEEE 802.15.7 standard.

## 3. IEEE 802.15.7 Overview

**Figure 5:**
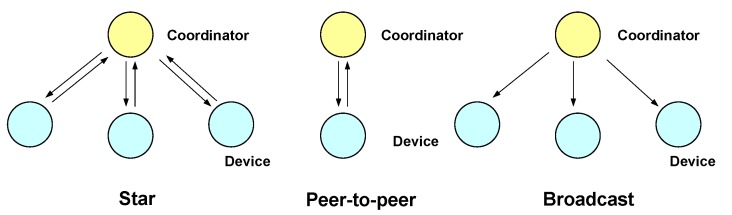
Network topologies defined in IEEE 802.15.7.

This standard defines a PHY and MAC layer for short range optical wireless communications using visible light, employing free space as propagation medium. Three network topologies are defined: star, peer-to-peer and broadcast. These topologies are shown in Figure 5, and their main characteristics are summarized as follows:

- **Star**: communication is made between several devices and one central controller, called the coordinator, which is also used as an illumination infrastructure. The rest of devices are typically mobile terminals powered by batteries.
- **Peer-to-peer**: allows two devices to communicate, provided they are within reach of each other. One of the devices acts as the communication coordinator
- **Broadcast**: one coordinator sends data to one or several devices in a uni-directional way.

### 3.1. PHY Layer

The IEEE 802.15.7 standard PHY layer is responsible for several functions, such as transmission and reception via the optical medium, activation and deactivation of the optical transceiver, and detection of the state of the transmission channel (idle/busy).

### 3.2. PHY Layer Operation Modes

The PHY layer supports three operation modes, which allow the use of different transmission rates and include support for different types of optical devices and operating environments:

- **PHY I**: intended for outdoor usage with low data rates (from 11.6 Kbps to 266.6 Kbps).
- **PHY II**: intended for indoor usage with moderate data rates (from 1.25 Mbps to 96 Mbps).
- **PHY III**: for applications with multiple optical transmitters and receivers working on different frequencies (colors). It uses the technique of modulation Color-Shift Keying (CSK) [34], with transmission rates from 12 Mbps to 96 Mbps.

Each operation mode of the PHY layer supports the use of different combinations of transmission rates, optical clock frequencies, modulation schemes, line codes, and error correction coding techniques. Each one of the aforementioned parameters settings is designated with an operation mode identifier or Modulation Coding Scheme Identifier (MCS-ID).

#### 3.2.1. Idle Patterns and Brightness Control

The operation of IEEE 802.15.7 devices of infrastructure type—such as the coordinator—must be compatible with its use as a lighting source. According to the standard, infrastructure devices must transmit idle patterns during the time intervals they receive data or do not have transmission activity, in order to maintain visibility, ensure brightness control, and avoid visible flicker to the human eye. The standard allows idle patterns to be in-band or out-of-band, in relation to the frequency spectrum of the optical carrier modulating signal, *i.e.*, according to the spectrum of the optical detector output signal. In-band idle patterns do not require changes in the frequency of the optical clock, and remain within the frequency range the receiver circuits can accept. Out-of-band idle patterns maintain visibility using lower clock frequencies (can even use direct current) and do not lie in the receiver’s modulation-domain.

### 3.3. MAC Layer

Among the functions of the IEEE 802.15.7 MAC, the task of regulating optical medium access is the most important, allowing either scheduled or random access transmissions. Four random access methods are defined: unslotted random access, slotted random access, unslotted CSMA/CA and slotted CSMA/CA.

#### 3.3.1. Superframe Structure

The MAC layer allows the use of a time based structure called superframe, whose characteristics are defined by the coordinator. The superframe is a timing pattern that is delimited by beacon frames. The coordinator transmits beacon frames at the beginning of each superframe to synchronize devices, identify the network and define the characteristics of the superframe. The superframe can have an active part and an inactive part. The active part is divided in 16 equally spaced intervals or time slots, during which all communications are performed. The inactive part allows devices to pass to an energy saving state. The active part is divided into a Contention Access Period (CAP), where devices compete with each other to access the channel using a random access procedure, and a Contention Free Period (CFP), intended for applications with latency and bandwidth requirements. In the CFP channel, access is centrally managed by the coordinator through the use of time slots called Guaranteed Time Slot (GTS). The structure of the superframe is defined by the values of several parameters, described below:

- The attribute *macBeaconOrder, BO*, describes the interval used by the coordinator to transmit beacon frames. *BO* and *aBaseSuperframeDuration* attributes define the time interval between beacon frames, *BI* (beacon interval, specified in clock cycles) according to the following expression:
  (6) $$\begin{array}{cc} BI=aBaseSuperframeDuration\times 2^{BO} & for:0\leq BO\leq 14\\ \mathrm{If}\;BO=15\;\text{the}\;\text{coordinator}\;\text{does}\;\text{not}\;\text{transmit}\;\text{beacon}\;\text{frames}. & \end{array}$$
  where *aBaseSuperframeDuration* is defined as the number of clock cycles that form the superframe when the attribute *macSuperframeOrder* (see below) is equal to 0.
- The attribute *macSuperframeOrder, SO*, defines the length of the active part of the superframe, including the beacon frame. The relationship between the value of *SO* and the duration of the active part of the superframe, *SD* (superframe duration, measured in clock cycles) is given by:
  (7) $$\begin{array}{cc} SD=aBaseSuperframeDuration\times 2^{SO} & for:0\leq SO\leq BO\leq 14\\ \text{If}\;SO=15\;\text{the}\;\text{frame}\;\text{does}\;\text{not}\;\text{have}\;\text{active}\;\text{part}. & \end{array}$$

The beacon frame is transmitted at the beginning of the first slot of the superframe without the use of any random access method. The CAP begins immediately after the beacon frame, and the CFP—if it exists—begins following the CAP and extends to the end of the active part of the superframe.

#### 3.3.2. Random Channel Access

When transmitting during the CAP of the superframe, the MAC layer uses the slotted version of the random access algorithm defined in the standard. This algorithm is implemented using units of time called backoff periods, where one backoff period is equal to *aUnitBackoffPeriod* clock cycles:

(8) aUnitBackoffPeriod=20opticalclockcycles

**Figure 6:**
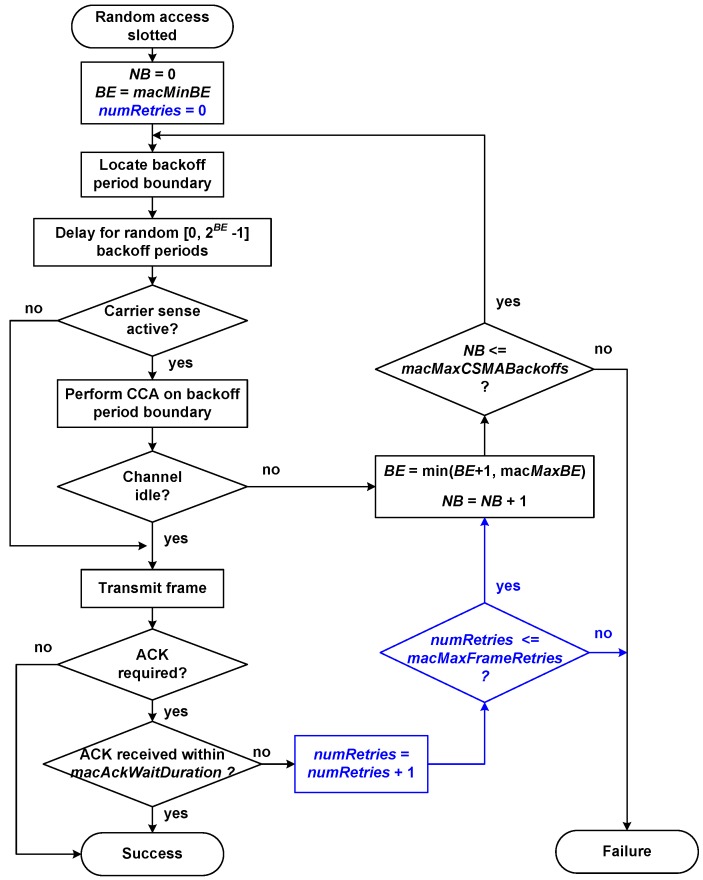
Random access algorithm implemented in our simulation model.

Figure 6 shows the flow chart of the slotted random access algorithm used in IEEE 802.15.7, which is briefly explained next. During every frame transmission attempt, each device maintains two variables:

- *NB* (Number of Backoffs): counts the number of times the medium access algorithm has made a backoff—random delay—during the current transmission attempt. This variable is initialized to zero at the beginning of each new frame transmission attempt.
- *BE* (Backoff Exponent): related to the number of backoff periods, a device shall wait before attempting to access/assess a channel. This variable is initialized to the value of macMinBE attribute.

Access procedure begins with the initialization of NB and BE variables. Next, backoff periods in the device are aligned with the beginning of the superframe. Then, the MAC layer shall delay for a random number of complete backoff periods in the range $\left[0,2^{BE}-1\right]$. If carrier detection is enabled, the PHY layer is requested to perform the CCA (Clear Channel Assessment) function to check if the channel is idle and available to transmit. If the PHY layer evaluates that the channel is busy, the MAC layer will increase variables *NB* and *BE*, ensuring that *BE* does not exceed the value of *macMaxBE* attribute. If *NB* is less than or equal to the value of the *macMaxCSMABackoffs* attribute, a new backoff delay will begin. If *NB* is greater than *macMaxCSMABackoffs*, the algorithm will terminate with a channel access failure status. If the PHY layer evaluates that the channel is available—or if the CCA is not active—the frame will be transmitted. After transmitting the frame, if enabled the use of acknowledgement (ACK), the MAC layer will wait for at most macAckWaitDuration optical clocks for the corresponding ACK. In case the ACK frame is received, the procedure will finish successfully. In case the ACK frame is not received, *NB* and *BE* variables will be increased to perform a new backoff, provided that NB is not greater than *macMaxCSMABackoffs*.

In our developed simulation model, we have added the necessary steps to the algorithm in order to verify that the maximum number of frames retransmissions limit is not exceeded, in accordance with clause number 5.1.7.5 of the standard. This clause specifies that when the ACK frame is not received, the data frame should be retransmitted, repeating this sequence up to a maximum of macMaxFrameRetries times. If a data transfer attempt fails a total of (1+macMaxFrameRetries) times, the MAC layer will finish the procedure with a frame transmission failure. To fulfill this requirement, we have added the numRetries variable, which accounts for the number of frame retransmissions, adding also the necessary steps to verify that the limit of *macMaxFrameRetries* retransmissions is not exceeded (marked in blue color in the flow chart).

## 4. Simulation Model

We built our simulator using OMNET++ simulation framework [35] from the model developed by [36], designed for sensor networks based on the IEEE 802.15.4 standard, due to the similarities that exist between the architectures of IEEE 802.15.7 and IEEE 802.15.4 standards.

### 4.1. Characteristics of Simulation Scenarios

Our developed simulation model has been designed with the following premises:

- We have chosen IEEE 802.15.7 star topology, due to its importance and wide range of applications.
- For the MAC layer, we opted to use the superframe, since it allows the use of both contention (CAP) and no contention (CFP) access methods. In addition, the use of the superframe enables devices to enter the energy save state during the idle period.

**Figure 7:**
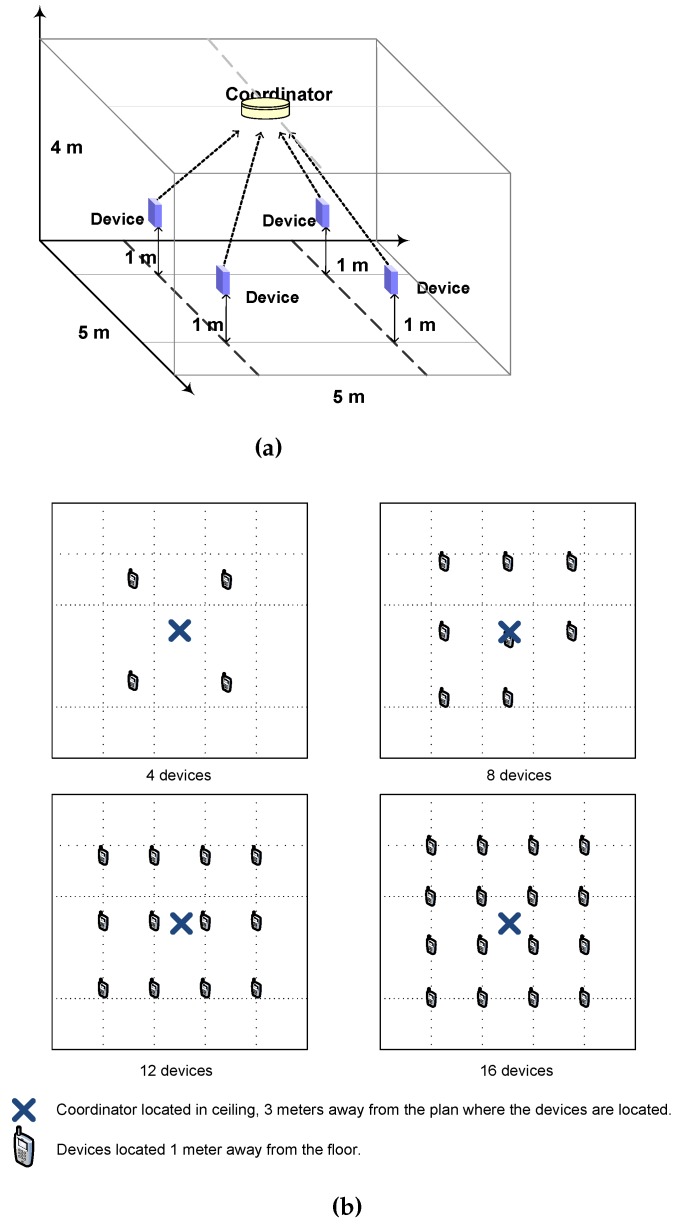
(**a**) Star network scenario with 4 devices in 4 m × 5 m × 5 m enclosure and (**b**) top-view layout for scenarios with 4, 8, 12 and 16 devices.

Our developed simulation model has been designed to evaluate the performance of uplink transmissions during the CAP of the superframe. For this purpose, we have defined four basic scenarios that differ in the number of devices, *N*, that communicate with the coordinator. We have defined scenarios with N=4,8,12and16 devices, respectively. In all scenarios, network nodes are contained in an enclosure of 4 m height by 5 m wide and 5 m long. The coordinator is located in the center of the ceiling and points towards the ground. Devices point to the coordinator and are located in fixed positions distributed according to a grid pattern on a plane situated at the height of 1 m above the ground. Figure 7a illustrates the simulated network layout of the scenario with four devices. Figure 7b shows the top view of scenarios with 4,8,12and16 devices. All scenarios share the same configuration parameters for PHY and MAC (taking into account that the orientation vector of each device changes with its position).

Next, we present the most important features of our simulation model, which are needed to adequately interpret the results obtained from the simulations performed.

### 4.2. Optical Channel Model

The transmission medium is modeled as free space without obstacles. We chose the directed LOS link configuration to model the optical signal propagation, requiring a line of sight between each device and the coordinator. We have considered the direct component of the received signal to calculate the power of the received optical signal, not considering the existence of reflections. According to the results presented in [20], at least 90% of total received optical power is direct light in VLC when using a receiver field of view (FOV) of 60${}^{{}^{\circ}}$. Therefore, to ensure the validity of our implemented model we have configured all optical receivers using the value of 60${}^{{}^{\circ}}$ for FOV. The adopted optical channel model facilitates reaching high transmission speeds, since the effects of multipath distortion on the optical signal are not considered. Among its drawbacks is the limitation in device mobility, since they must be permanently oriented to the coordinator. To meet this requirement, devices in our simulation model are able to automatically point to the coordinator. Calculation of received power considering only direct component of the signal has the additional benefit of improving the efficiency of the implemented simulation model. The computational load required to run simulations of scenarios with multiple nodes including the functionality of different layers of the architecture is significantly reduced.

### 4.3. Application Layer Traffic Characterization

In order to evaluate the performance of uplink communications in our simulation model, the application layer on each device generates data messages destined to the coordinator. The time intervals between generated messages follow an exponential probability distribution. Each message is encapsulated in the MAC Service Data Unit (MSDU) of one data frame. All devices in all scenarios use the same application layer settings. This way, in a scenario with *N* devices employing an application message size of *L* bits and average time between messages of $t_{s}$ seconds, total offered load $T_{L}$ normalized to the physical layer transmission rate $R_{b}$ is:

(9) $$T_{L}=N\cdot \frac{L}{t_{s}\cdot R_{B}}\left(\%\right)$$

## 5. Simulation Results Analysis

In this section, we present and analyze the results obtained from simulations made with our implemented model. The effects of the hidden node problem are evaluated by comparing simulation results when there are not hidden nodes with results when there are hidden nodes in the network. To ensure the reliability of the results, several independent simulations have been replicated for the same set of input parameters, averaging output result values. Simulation time was at least 400 s per replica.

### 5.1. Simulation Parameters

This section presents the main configuration parameters used in the developed simulation model.

#### 5.1.1. PHY Layer Simulation Parameters

Table 1 shows the main configuration parameters of PHY layer used in all simulation scenarios. We selected the PHY II operating mode, intended for both indoor and outdoor environments, using MCS-ID number 16, since support for the minimum clock and data rates for a given PHY is mandatory. Optical transmission power of devices is of 30 mW, in accordance with the limitations of power consumption in battery-powered mobile terminals. According to the optical channel model used, transmitters’ directivity is characterized by its half power angle, $\theta_{1/2}$, while receivers’ directivity is defined by its FOV. According to [20], both parameters are assigned a value of 60${}^{{}^{\circ}}$, to ensure validity of the implemented channel model, since the calculation of received optical power takes into account only the direct component of the signal. In order to simplify the calculation process of the model, the values used for the concentrator gain (G) and the transmission coefficient of the optical filter (T) are considered to be constants, so they do not depend on the angle of incidence (ψ).

**Table 1:** Main configuration parameters of the physical (PHY) layer.

Parameter	Value
Transmission rate	1.25 Mbps
Optical clock rate	3.75 MHz
Coordinator optical transmission power	1500 mW
Devices optical transmission power	30 mW
Half Power Angle $\theta_{1/2}$	60${}^{{}^{\circ}}$
Field of Vision (FOV)	60${}^{{}^{\circ}}$
Photo detector area (A)	1 cm${}^{2}$
Photo detector responsivity (R)	0.54 A/W
Optical concentrator gain (G)	15
Optical filter transmission coefficient (T)	1
Clear Channel Assessment (CCA) function duration	8 clock cycles
*aTurnaroundTime-TX-RX*	0 clock cycles
*aTurnaroundTime-RX-TX*	8 clock cycles

The values selected to define the rest of characteristics of optical transmitters and receivers are commonly used values to characterize VLC transmitters and receivers, similar to those used in [37,38,39]. Lastly, the contribution of noise in the channel (thermal and shot noise) has been considered as negligible in all simulations. Thus, the implementation of the simulation model is simplified, ensuring also that failures in frame transmissions are only caused by collisions (simultaneous transmissions on the network). In this way, noise level or transmission errors do not affect the simulation results.

#### 5.1.2. MAC Layer Simulation Parameters

Regarding the configuration of the superframe, both BO (macBeaconOrder) and SO (macSuperframeOrder) parameters are set with the same value, so that there is no inactive period in the superframe. Since this work is intended to evaluate the performance of uplink communications during the CAP, we decided not to assign any slot of the superframe to the CFP. Thus, the entire superframe is available to perform transmissions using the CSMA/CA based slotted random algorithm. The value assigned to both the BO and SO parameters is:

(10) BO=SO=9

We performed several simulations using larger values for both *BO* and *SO* parameters, up to the maximum limit allowed by the standard when the superframe is used (*SO = BO =* 14). We checked that network performance tends to improve as the value used for both BO and BO increases. The rest of the MAC layer configuration parameters were chosen according to the default values defined in the standard. Table 2 shows the values used for the main configuration parameters of the MAC layer.

**Table 2:** Main configuration parameters of the medium access control (MAC) layer.

Parameter	Value
*macMaxBE*	5
*macMinBE*	3
*macMaxCSMABackoffs*	4
*macMaxFrameRetries*	3

#### 5.1.3. Application Layer Simulation Parameters

Application message sizes of 512 bytes, 1024 bytes and 1500 bytes were used for simulations (same size for all devices). The largest message size—1500 bytes—was selected according to the maximum transmission unit of the Ethernet standard. Simulation results did not vary significantly depending on application message size, since variation margin was below 5%. Hence, we decided to present here only the results obtained with the intermediate message size of 1024 bytes.

The IEEE802.15.7 standard limits the frame size (PHY layer SDU) to 65,535 bytes when PHY II and PHY III is used. When PHY I is used, the maximum frame size is 1023 bytes.

### 5.2. Simulation Metrics Evaluated

Network performance was evaluated by means of the following metrics:

- End-to-end goodput: calculated as the average number of bits of application layer messages received per unit time in the coordinator. This value is normalized and expressed as a percentage of the transmission rate used in the PHY layer.
- End-to-end message loss rate: percentage of application messages generated in devices not received by the coordinator. Packets are not received by the coordinator due to the following reasons: because there is an overflow in the queue storing the messages to be transmitted by the MAC layer, due to channel access failures (when *macMaxCSMABackoff* limit is exceeded), or due to frame transmission failures (when *macMaxFrameRetries* limit is exceeded).
- CSMA/CA statistics: obtained from the execution of the random access algorithm in each device. Example statistics are: the percentage of successfully transmitted frames, the percentage of collisions (ACK frames not received), the percentage of channel access failures and the percentage of frame transmission failures.
- Energy consumption: calculated from consumed current by the optical transceiver in each device. Consumption depends on the states through which the transceiver passes along simulation time.

### 5.3. Performance of Uplink Communications with No Hidden Nodes

In order to exclude the existence of hidden nodes in the network due to the directivity of the optical transmitters and receivers, the simulated channel model was modified to remove all directivity characteristics in them. Additionally, devices were configured with an optical transmission power of 150 mW to prevent the occurrence of hidden nodes due to the limitation on the optical signal coverage. Using the described channel model, we verified that each and every device in the network detects all transmissions from all other devices in all of the simulation scenarios. Hence, the existence of hidden nodes is discarded. Although the employed channel model does not match any link configuration used in VLC networks, it allows establishing the simulation conditions that guarantee the non-existence of hidden nodes in the network. The simulation results achieved with these conditions will be compared with the results of simulations performed employing the LOS channel model, in order to assess the impact of hidden nodes on network performance.

#### 5.3.1. Goodput and Message Loss Rate

**Figure 8:**
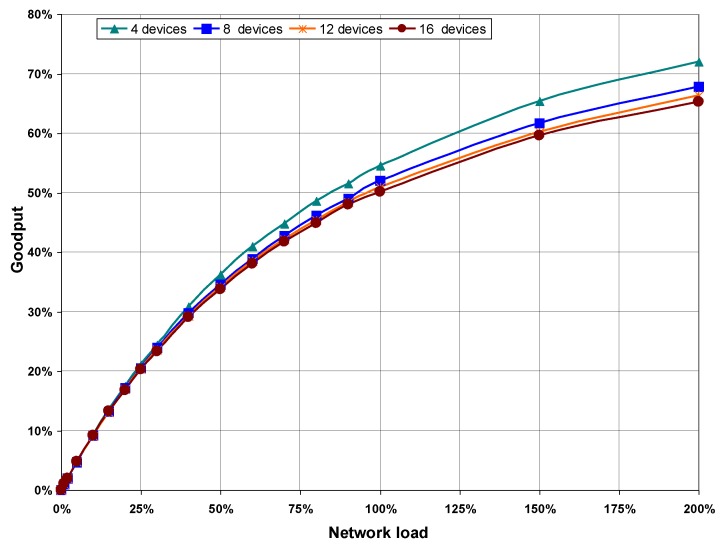
Goodput as a function of network load, with no hidden nodes.

Figure 8 shows goodput values obtained from simulations performed in the four scenarios, plotted as a function of network load. As can be seen, the goodput increases as network load does, although it is noted that higher load values imply lower goodput on scenarios with more devices on the network. This is because the more devices that are in the network, the higher the number of collisions for the same network load, as can be seen in the statistics obtained from the execution of the CSMA/CA algorithm shown in Table 3 (see Section 5.3.2 below).

#### 5.3.2. CSMA/CA Statistics

In order to analyze how the CSMA/CA algorithm affects goodput and message loss rate, detailed performance statistics have been obtained in each device. Table 3 shows statistical results for scenarios with four and 16 devices, obtained from three network load values: 10%, 50% and 200%. Values of statistics shown were calculated by averaging the results from all the devices in the network. Table 3 shows the following statistical results:

- Successfully transmitted frames: percentage of data frames which have been transmitted by the device and acknowledged by the coordinator. This percentage refers to the total number of data frames that MAC layers in the device attempts to transmit.
- Channel access failures: percentage of dropped frames by MAC layers in the device because the CSMA/CA algorithm has failed due to the maximum number of backoffs (*macMaxCSMABackoffs*) being exceeded. This percentage refers to the total number of data frames that MAC layers in the device attempts to transmit.
- Frame transmission failures: percentage of dropped frames by MAC layer in device because the CSMA/CA algorithm has failed due to the maximum number of frame transmission retries (*macMaxFrameRetries*) being exceeded. This percentage refers to the total number of data frames that MAC layers in the device attempts to transmit.
- Collisions: percentage of transmitted data frames for which no acknowledgment frame has been received, due to transmission failure. According to simulation conditions, the only reason that can cause a frame transmission failure is a collision. This percentage refers to the total number of data frames transmitted by MAC layers in the device.

**Figure 9:**
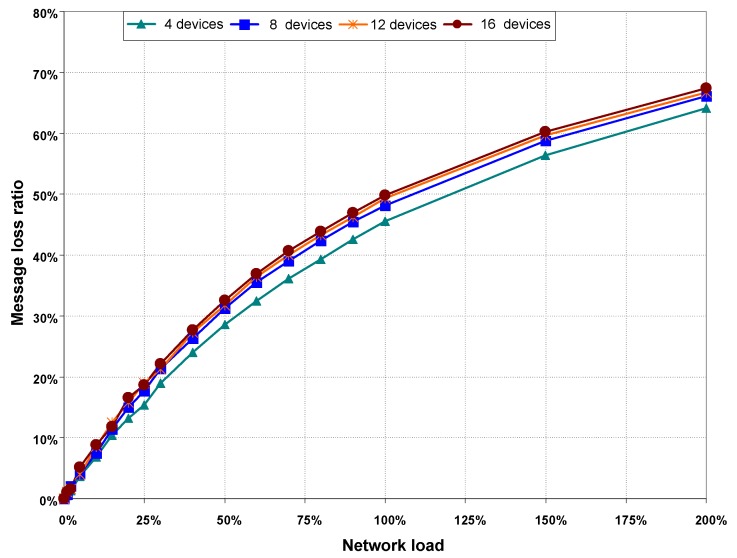
Message loss rate as a function of network load, without hidden nodes.

Table 3 shows that the percentage of successfully transmitted frames decreases as both network load and the number of devices increase, which demonstrates the results of message loss rate shown in Figure 9. Values shown in Table 3 point out that in the absence of hidden nodes, all failures in the CSMA/CA algorithm are recorded in the column “Channel access failures”, due to the *macMaxCSMABackoffs* limit being exceeded. The number of frame retransmissions caused by collisions is not high enough to cause the CSMA/CA algorithm to fail due to the maximum limit of retransmissions (*macMaxFrameRetries*) being exceeded.

**Table 3:** Carrier sense multiple access with collision avoidance (CSMA/CA) statistics with no hidden nodes.

N		Successfully		Frame	
(Number of	Network	Transmitted	Channel Access	Transmission	Collisions
Devices)	Load	Frames	Failures	Failures	
	**10%**	93.2%	6.8%	0%	0.5%
4	**50%**	71.8%	28.2%	0%	2.9%
	**200%**	38.2%	61.8%	0%	11.0%
	**10%**	91.1%	8.9%	0%	0.8%
16	**50%**	67.4%	32.6%	0%	3.8%
	**200%**	32.8%	67.2%	0%	14.2%

Values shown in Table 3—as well as results obtained from the rest of network loads and scenarios—show that the percentage of collisions increases with both network load and the number of devices in the network. Nevertheless, collision rates with no hidden nodes stays at relatively low values, below 15% in all cases. In conclusion, when there are not hidden nodes in the network, the main cause of channel access failures is due to the transmission medium being sensed as busy when CCA function is called in the CSMA/CA algorithm.

#### 5.3.3. Energy Consumption

Energy consumption results have been calculated from average current consumption by transceivers in devices during the entire simulation time. Due to present unavailability of VLC transceiver consumption specification data, simulations have been carried out with consumption values corresponding to the IRMT6452 [40] optical infrared transceiver. This transceiver conforms to the specifications defined by the Infrared Standar Associated [41] and combines an LED, a photodiode and associated circuitry. Absolute values for energy consumption obtained this way should be reasonably representative of real consumption experienced in a VLC network. Nevertheless, according to the objectives of this paper, energy consumption comparisons can be made depending on whether or not there are hidden nodes in the network.

**Figure 10:**
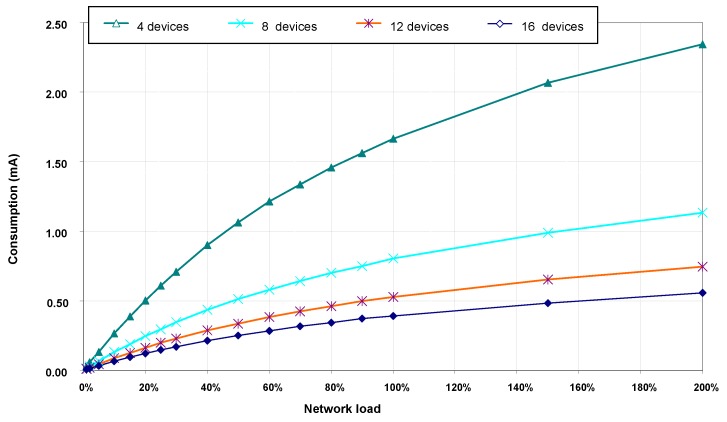
Average current consumption per device, without hidden nodes.

Figure 10 shows average values of accumulated current consumption by transceivers in each device during simulation time. Energy consumption in each device is higher in scenarios with fewer devices because network load is evenly distributed among all network devices. Thus, with a given load value, the number of transmissions performed by each device is higher in scenarios with fewer devices. Figure 10 also shows that energy consumption increases with network load, but not linearly with it. As indicated before, in the absence of hidden nodes, the time spent by devices doing backoffs and calling the CCA function increases with network load. In these states, the transceiver consumption is lower than in the transmission state.

### 5.4. Performance of Uplink Communications with Hidden Nodes

This section presents simulation results obtained by using the optical channel model based on directed LOS link configuration. Directed LOS link configuration results in the occurrence of hidden nodes in the network, due to the directivity of transceivers and limited optical transmission power of mobile devices (30 mW). It was found that, in all simulation scenarios, devices can only communicate with the coordinator. None of the devices in the network can detect the transmissions of any other device. Hence, in VLC networks with star topology employing directed LOS link configuration, all devices are hidden nodes to each other.

#### 5.4.1. Goodput and Message Loss Rate

**Figure 11:**
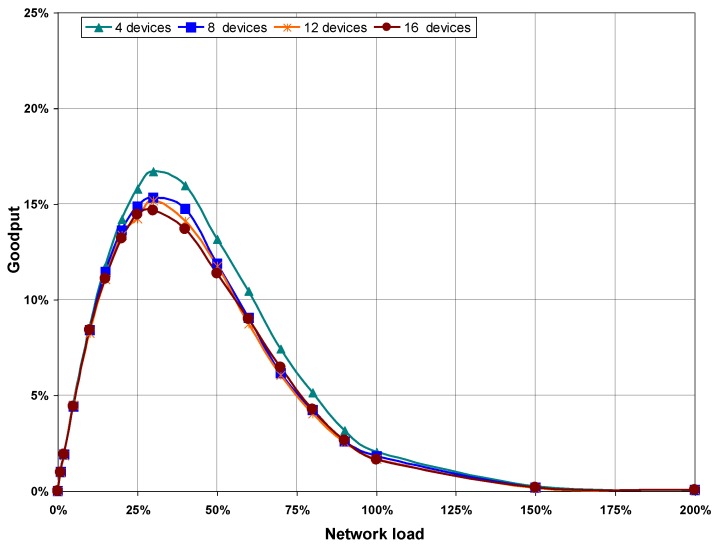
Goodput as a function of network load, with hidden nodes.

Figure 11 shows goodput results as a function of network load. The maximum goodput does not reach 20% of the transmission rate in any of the scenarios, which is well below the maximum goodput achieved when there are not hidden nodes in the network (between 65% and 72%). Furthermore, Figure 11 shows how the goodput drops abruptly when network load is higher than 30%, because of the increasing number of collisions.

**Figure 12:**
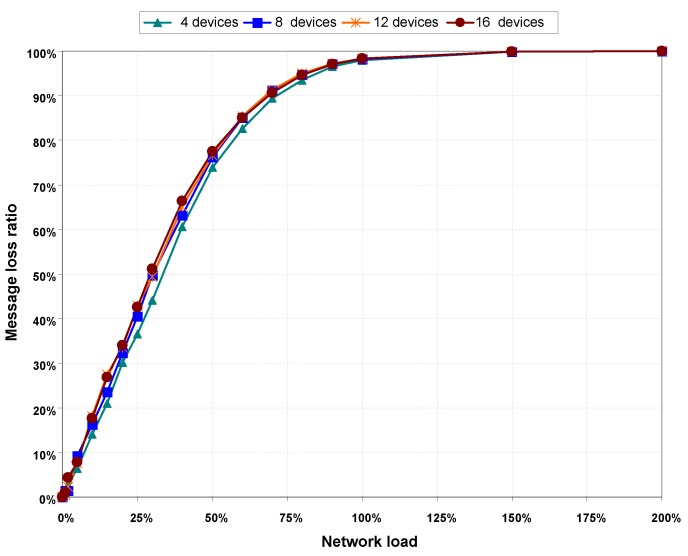
Packet loss rate, with hidden nodes.

Goodput results are validated with message loss ratio values presented in Figure 12. This Figure shows a rapid increase in message loss ratio with network load, compared to the results obtained when there are no hidden nodes in the network. Comparing the graphs of goodput in Figure 11 and message loss rate in Figure 12, it can be seen that goodput is practically zero and message loss rate is almost 100% for network load values greater than 150%.

#### 5.4.2. CSMA/CA statistics

Table 4 shows the results of the CSMA/CA statistics obtained with hidden nodes. As with the case with the statistics obtained when there are not hidden nodes, the percentage of successfully transmitted frames decreases as the load and the number of nodes in the network increase. However, in the presence of hidden nodes, the decrease in the percentage of successfully transmitted frames is much more pronounced, becoming virtually 0% with a 200% network load on all scenarios. The sharp decrease of successfully transmitted frames is caused by the rapid increase in the number of collisions, whose percentage turns out to be above 90% with network loads higher than 50%.

Due to the high percentage of collisions produced with hidden nodes, the whole failures produced in the CSMA/CA algorithm are accounted in the column “Frame transmission failures”, produced when the maximum retransmission number (*macMaxFrameRetries*) is exceeded. This makes a lot of difference with the statistics obtained without hidden nodes, in which all CSMA/CA failures are accounted in the column “Channel access failures”, produced when the limit of *macMaxCSMABackoffs* is exceeded.

**Table 4:** CSMA/CA statistics, with hidden nodes.

N		Successfully		Frame	
(Number of	Network	Transmitted	Channel Access	Transmission	Collisions
Devices)	Load	Frames	Failures	Failures	
	**10%**	85.9%	0%	14.1%	41%
4	**50%**	28.61%	0.03%	71.36%	91.2%
	**200%**	0.1%	0%	99.9%	99.9%
	**10%**	82%	0%	18%	48.2%
16	**50%**	22.73%	0.02%	77.25%	93.3%
	**200%**	0.02%	0%	99.98%	99.9%

#### 5.4.3. Energy Consumption

**Figure 13:**
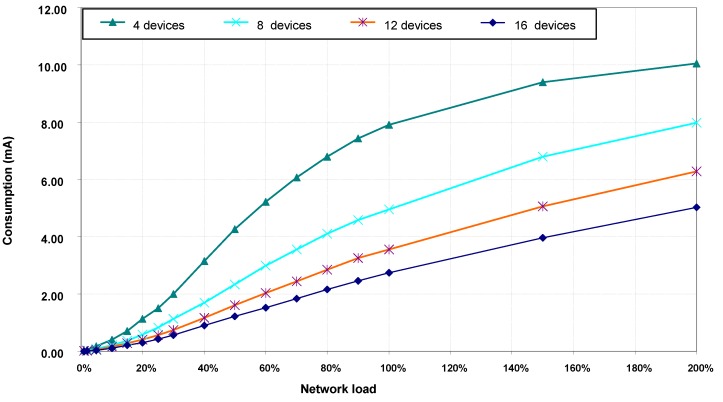
Average current consumption per device, with hidden nodes.

Figure 13 shows the average current consumption by transceiver in each device during simulation time. Compared to consumption obtained without hidden nodes in the network, consumption values with hidden nodes remain far superior in almost all load values and all of the scenarios. The largest increase in current consumption with hidden nodes is due to the increase in the number of frames’ retransmissions because of the collisions produced.

Differences between current consumption obtained in the absence and presence of hidden nodes are more obvious when using, as a metric, the values for total consumed current per MAC payload byte transferred on the network; that is, the current drawn by all devices which is needed to successfully receive an application message byte in the coordinator. Table 5 shows results of current consumption per payload byte, for scenarios of four and 16 devices and load values of 50%, 100% and 200%. Values in Table 5 show an increasing consumption per byte in both scenarios as network load increases, both in the absence and presence of hidden nodes. However, with hidden nodes and network loads greater than 100%, current consumption per payload byte reaches values of magnitude higher than values obtained without hidden nodes. With hidden nodes in the network, the increase in the number of collisions with transmission load has a double effect in current consumption per payload byte. On one hand, current consumption is significantly increased, as Figure 13 shows. On the other hand, the number of received messages in coordinator is drastically reduced, as shown in message loss ratio values displayed in Figure 12.

**Table 5:** Comparison of current consumption per payload byte.

N		Current Consumption	Current Consumption
(Number of	Network Load	with no Hidden Nodes	with Hidden Nodes
Devices)		(mA/byte)	(mA/byte)
	**50%**	$1.89\times 10^{-7}$	$2.08\times 10^{-6}$
4	**100%**	$1.96\times 10^{-7}$	$2.51\times 10^{-5}$
	**200%**	$2.09\times 10^{-7}$	$1.45\times 10^{-3}$
	**50%**	$1.91\times 10^{-7}$	$2.76\times 10^{-6}$
16	**100%**	$2.00\times 10^{-7}$	$4.27\times 10^{-5}$
	**200%**	$2.19\times 10^{-7}$	$2.62\times 10^{-3}$

## 6. Solution Proposal to the Hidden Node Problem

In this section, we present a solution proposal to the hidden node problem appearing in uplink communications made during the CAP of the superframe in the IEEE 802.15.7 standard. The proposed solution is based on the solution presented in [42] for networks based on radio frequency using CSMA channel access method, with a central node responsible of managing all transmissions made by the rest of the nodes. According to the model presented in [42], the central node starts sending a signaling tone—called *busy tone*—when it detects a transmission from one of the nodes in the network. The *busy tone* is received by all nodes in the network, and notifies them that there is an ongoing transmission being made. Results presented by the authors of [42] show that their solution performs almost as well as CSMA without hidden terminals. The solution we present in this work is based on a [42] model principle, applied to IEEE 802.15.7 networks with star topology. In our proposed solution, the coordinator is responsible for sending a signaling pattern—analogous to the *busy tone*—to indicate it is receiving a transmission coming from one of the devices in the network.

### 6.1. Model Description

**Figure 14:**
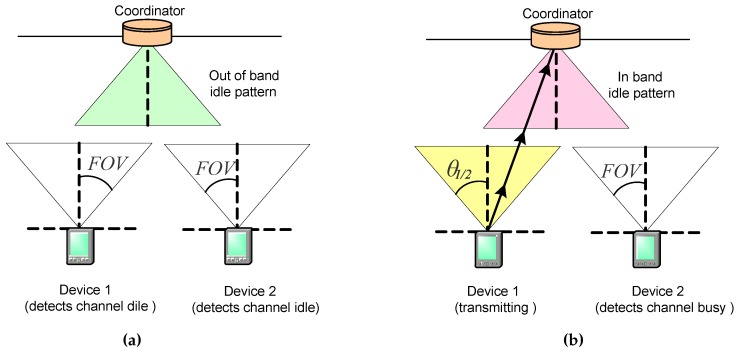
Transmission of out-of-band (**a**) and in-band (**b**) idle patterns by the coordinator for signaling transmission activity.

As described in Section 3.2.1, the IEEE 802.15.7 standard indicates that sending an idle pattern is required for the coordinator during idle or receive operation to ensure continuous illumination. The solution we propose is based on selecting the appropriate type of idle pattern sent by the coordinator, depending on whether or not it is receiving transmissions from the devices, as such it is described below:

- When the coordinator does not receive transmissions from the devices, it sends an out-of-band idle pattern. Thus, devices wishing to make uplink transmissions during CAP detect the channel idle when they invoke the CCA function during CSMA/CA procedure. Figure 14a shows an example of this situation: coordinator sends an out-of-band idle pattern and devices 1 and 2 detect the channel idle, so they can continue to transmit.
- When the coordinator receives a transmission from one or more devices, it sends an in-band idle pattern. Thus, devices wishing to make uplink transmissions during CAP detect the channel as busy when they invoke the CCA function during CSMA/CA procedure. Figure 14b shows an example of this situation: coordinator sends an in-band idle pattern when receiving the transmission from device 1, and device 2 detects the channel as busy.

Our proposed solution has the advantage of not requiring modifications to the specifications defined in the IEEE 802.15.7 standard for PHY and MAC layers in devices and coordinators. However, according to the results published in some papers, such as [43], simultaneous transmission of optical signals on both directions— idle patterns in downlink direction and frames in uplink direction—may produce mutual interference. Therefore, to minimize such interference, it is necessary to configure PHY layers in devices and coordinators in order to employ different wavelength bands for each transmission direction. According to the IEEE802.15.7 standard, the PHY layer allows the selection of wavelength bands by means of the so called Band Plan Ids, which indicate the codes corresponding to the wavelengths containing the spectral peak for the transmitted frames. Band Plan Ids are indicated in the PHY header, and may be used by the receiver for optimizing its performance in order to minimize the interference.

### 6.2. Simulation Results

To check the effectiveness of our proposed solution, we performed several simulations by applying it to our four defined scenarios. We employed the directed LOS channel model, maintaining the same simulation conditions we used when evaluating network performance with hidden nodes. Simulation results obtained with our solution are practically identical to those obtained from the simulations performed without hidden nodes in the network, for all scenarios and the whole network load values.

**Figure 15:**
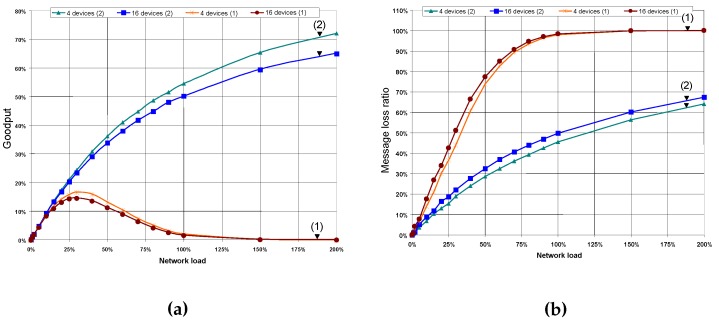
Comparative the Goodput (**a**) and the Message loss rate (**b**) among the simulation scenarios labeled as (1) when a signaling pattern is used to indicate that a device is transmitting and the scenarios labeled as (2) when a signaling pattern is not used. The results shown are for the scenarios with four and 16 devices.

To confirm the validity of our results, Figure 15 shows a comparative for goodput and message loss ratio values obtained from the simulation scenarios with hidden nodes in the network. In the first experiment labeled as (1) none of the devices in the network can detect transmissions of any other devices, therefore collisions are produced. Since no signaling mechanism is used to avoid collisions, maximum goodput does not reach 20%, see Figure 15a, and message loss rate increases rapidly, see Figure 15b.

Results of the second experiment labeled as (2) have been obtained by applying our proposed solution. The coordinator sends a signaling pattern when it detects a transmission coming from one of nodes in the network. Thus, the rest of nodes in the network know that the channel is not free. In this case, goodput results are better than the results obtained in experiment (1). In addition, message loss rate is lower. The performance of the VLC network improves when our mechanism is used to avoid the hidden node problem. Results obtained in (2) are similar to those obtained from simulations performed without hidden nodes, see Figure 8 and Figure 9.

## 7. Conclusions

Since it was published in 2011, the IEEE 802.15.7 standard has become one of the most important initiatives to normalize and deploy the use of VLC in WPAN. Though the implantation of IEEE 802.15.7 based networks must face several technical challenges, among which are the difficulties in uplink transmissions (from devices to coordinators), including limitations in mobile device transmission power imposed by LED power consumption, and mobility restrictions caused by the necessity of keeping a proper orientation in devices.

Additionally, uplink transmissions made by means of the CSMA/CA method in the IEEE 802.15.7 standard are affected by the hidden node problem, whose effects have been evaluated and analyzed in this research work. We have evaluated the hidden node problem by simulating a star network employing the directed LOS channel model. Under these conditions, we verified that all devices are hidden nodes in all of our modeled scenarios, since none of the devices are capable of detecting the signal transmitted by the rest of them. Our simulation results make evident a severe degradation in network performance produced as a consequence of the hidden node problem. Performance degradation is proven by obtained values for the analyzed metrics, like end-to-end goodput, message loss ratio, percentage of collisions, and energy consumption.

Actual performance degradation may depend on specific characteristics of optical transmitters and receivers used in devices. The use of less directive transmitters and receivers could partially mitigate the effects of the hidden node problem in networks employing a diffuse link configuration. In any case, limitations in optical signal coverage—due to restrictions in transmission power in devices—demand not ignoring the effects of the hidden node problem, regardless of the link configuration employed.

In conclusion, the hidden node problem has to be included as one of the difficulties to be solved in uplink transmissions made by using the CSMA/CA method in IEEE 802.15.7 networks. Therefore, as part of this research work, we elaborated on a solution proposal to the hidden node problem, which has the advantage of not requiring modifications in the specifications defined in the standard for PHY and MAC layers in devices and coordinators.

Our proposed solution is based on the use of the idle patterns sent by the coordinator, during idle or receive operation. Appropriate selection of idle pattern type—in-band or out-of-band—sent by the coordinator can indicate if it is receiving transmissions from devices in the network. Simulation results obtained with our solution demonstrate its high degree of effectiveness, since it allows obtaining identical performance values to those obtained from the simulations performed without hidden nodes in the network.
